# Magnetic Molecularly Imprinted Polymers with Hydrophilic Shells for the Selective Enrichment and Detection of Rosmarinic Acid in Aqueous Extraction

**DOI:** 10.3390/plants14010056

**Published:** 2024-12-27

**Authors:** Yanhui Wang, Linlin Yan, Guangyao Zheng

**Affiliations:** Key Laboratory of Biomass Energy and Material, Jiangsu Province, Key Laboratory of Chemical Engineering of Forest Products, National Forestry and Grassland Administration, National Engineering Research Center of Low-Carbon Processing and Utilization of Forest Biomass, Jiangsu Co-Innovation Center of Efficient Processing and Utilization of Forest Resources, Institute of Chemical Industry of Forest Products, CAF, Nanjing 210042, China; wangyanhui0718@163.com (Y.W.); zhguya@sina.com (G.Z.)

**Keywords:** hydrophilic magnetic molecular imprinted polymer, rosmarinic acid, aqueous extraction

## Abstract

Rosmarinic acid (RA) is a natural active compound widely found in many plants belonging to the family of *Lamiaceae*, *Boraginaceae*, and so on, which has various important bioactivities, including being anti-oxidative, anti-inflammatory, antiviral, etc. Herein, novel hydrophilic magnetic molecularly imprinted polymers (HMMIPs) with a regular core-shell structure were successfully developed using RA as a template molecule, acrylamide (AM) as a functional monomer, N-N ’methylenebisacrylamide (MBA) as a cross-linking agent, and water as the porogen. After a series of characterization and adsorption performance analyses, it was found that HMMIPs are hydrophilic with an adsorption capacity of 8.012 ± 0.54 mg/g, an imprinting factor of 3.64, and a selectivity coefficient of 2.63~2.91. Furthermore, the HMMIPs can be rapidly separated from other components under the influence of external magnetic fields. The HMMIPs were employed for the determination of RA present in the *Perilla frutescens* and *Rosmarinus officinalis* aqueous extract with recoveries of 88.2~107.3%. These results indicated that HMMIPs of RA have the benefits of straightforward operation, rapid adsorption, and high selectivity, rendering it an appropriate way for the expedient and selective isolation of RA in an intricate matrix.

## 1. Introduction

Rosmarinic acid (RA) is an ester of 3,4-dihydroxyphenyllactic acid and caffeic acid [1]. It is commonly found in several medicinal plants of the *Lamiaceae* family and in traditional Chinese medicine [2]. Up to now, more than 240 plant species have been screened for the presence of rosmarinic acids [3]. RA has multiple biological activities, such as being antioxidant [4], anti-inflammatory [5], antibacterial [5], anti-tumor [6], and anti–mutagenic [7]. Therefore, the extraction process and extract have become topical issues. The extraction of RA is a challenging process due to its susceptibility to degradation, particularly in environments characterized by elevated temperatures, direct sunlight, and aqueous solutions with a high pH [8]. RA was first separated and named from rosemary by two Italian chemists, Scarpati and Oriente (1958) [9]. To date, a number of methods have been employed to achieve the preparative separation and purification of high-purity RA, including high-speed counter-current chromatography (HSCCC) [10], biotechnological production [11], and total synthesis [8]. However, the existing techniques are associated with a number of drawbacks, including the use of organic solvents, the cost of media, the time required for procedures, and the necessity for specialized instruments, which enhance process efficiency. Thus, the selective extraction of RA from the complex matrix to its quantification is an emergency.

Recently, a prominent approach to the selective extraction of bioactive chemicals entails a complex matrix, which is called molecular imprinting technology (MIT) [12,13]. The principles of molecular blotting have been previously described in numerous articles and are not reiterated here [14]. The most commonly employed methods include suspension polymerization, precipitation polymerization, and surface imprinting techniques [15]. As MIT has advanced, researchers are intrigued by the possible synergies between the technology of surface molecular imprint and magnetic separation. The formation of a core and shell structure within polymers enables the creation of magnetic molecular imprinted polymers (MMIPs), which exhibit a distinctive form and a substantial specific surface area [16]. MMIPs are synthesized by depositing thin imprinting layers on magnetic nanomaterial surfaces [17]. The imprinting layers are characterized by binding sites that are perfectly connected with the three-dimensional structure of the template, enabling the selective recognition of template molecules within a complex matrix [18]. Furthermore, the imprinted sites on magnetic nanoparticles facilitate template removal completely, rapid mass transfer, and high-speed separation, which are advantageous for analytical applications [19]. Zahara et al. [20] presented the selective extraction of RA via the molecular imprinting technique. The polymer displayed an excellent degree of selectivity toward RA, which represents a highly promising alternative to the conventional extraction techniques currently employed in the selective extraction of RA from the matrix. Alipour et al. [21] have synthesized RA-MIPs by employing an ultrasonic-assisted dispersive solid-phase extraction approach for the extraction of RA as the natural antioxidative species from the extract of *Salvia officinalis* leaves. The selectivity studies showed that, compared to other compounds such as gallic acid, caffeic acid, and pyrocatechol, the template molecule (i.e., RA) is bound selectively and with high affinity to the MIPs sorbent. However, the mentioned polymers have been prepared in organic solvents that are incompatible and poorly hydrophilic, which may lead to reduced extraction efficiency for RA in an aqueous matrix. The incorporation of hydrophilic functional monomers represents a prominent avenue for addressing the issue. At present, the most commonly use hydrophilic functional monomers include *β*-cyclodextrin, 2-hydroxyethyl methacrylate, acrylamide (AM), and functional monomers with hydrophilic properties that have been modified to enhance their characteristics. The cross-linking agent choosing the N-N′ methylenebisacrylamide (MBA), strong hydrophilicity, is an ideal raw material. It is our understanding that the use of AM as a functional monomer and MBA as a cross-linking agent in the development of MMIPs for the targeted adsorption of RA from an aqueous matrix has not yet been reported. The aim of the work is to investigate the hydrophilic magnetic molecular imprinted polymers (HMMIPs) and couple it with high-performance liquid chromatography (HPLC) for the selective enrichment and quantification of RA in plant aqueous extract. The synthesis of RA-HMMIPs in water was conducted using the reversible addition–fragmentation chain transfer (RAFT) method, with Fe_3_O_4_ employed as the carriers, RA as the template molecule, AM as the functional monomer, and MBA as the cross-linking agent. A comprehensive investigation was conducted into the synthesis conditions of RA-HMMIPs, along with the analysis of the physical–chemical properties and the conditions of adsorption. Furthermore, a methodology has been devised that combines the RA-HMMIPs with HPLC as a detection apparatus for purification and determination.

## 2. Results

### 2.1. Optimization of Synthesis Conditions for RA-HMMIPs

The strength and quantity of recognition sites can be determined by functional monomers interacting with template molecules [22]. Therefore, functional monomers were the key factor influencing polymer adsorption quantity. The functional monomers chosen for this experiment were acrylamide (AM), methacrylic acid (MAA), and 4-vinylpyridine (4-VP). It can be seen from Table 1 that the adsorption capacity of the RA-HMMIPs synthesized with methacrylic acid as a functional monomer is significantly lower than the others, presumably because the alkaline functional monomer is more likely to combine with RA to form a stable polymer. The adsorption capacity of the polymers prepared was close by AM and 4-VP. However, when AM was used as a functional monomer, the adsorption capacity was the highest (3.53 ± 0.42 mg/g) at a 1:6(template molecule: functional monomer) level. So, AM was selected as the functional monomer for subsequent experiments.

### 2.2. Characterization of RA-HMMIPs

**Figure 1:**
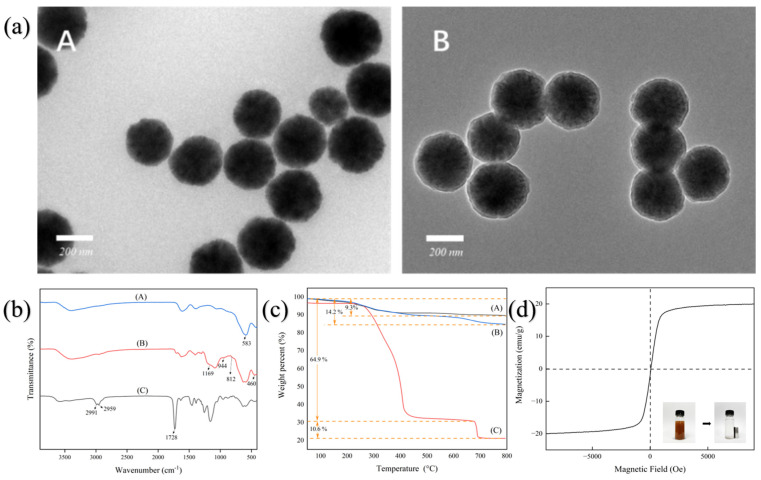
The characterization of RA-HMMIPs. Image (**a**): (**A**) The TEM image of Fe_3_O_4_ nanoparticles. (**B**) The TEM image of Fe_3_O_4_@SiO_2_-RAFT. Image (**b**): FT-IR spectra. Curve A. Fe_3_O_4_, Curve B. Fe_3_O_4_@SiO_2_-RAFT, Curve C. RA-HMMIPs. Image (**c**): The TGA curves. Curve A: Fe_3_O_4_, Curve B: Fe_3_O_4_@SiO_2_-RAFT, Curve C: RA-HMMIPs. Image (**d**): The VSM curves of RA-HMMIPs.

The morphology of the polymer was measured using a transmission electron microscope (TEM). As can be observed in Figure 1a(A), the image of the Fe_3_O_4_ nanoparticles reveals a relatively uniform spherical structure. Figure 1a(B) shows the morphological structure of the Fe_3_O_4_@SiO_2_-RAFT, which clearly shows that the thickness of the shell membrane of the core-shell structure increases slightly. It was proved that Fe_3_O_4_@SiO_2_-RAFT nanoparticles were successfully prepared and could be used for subsequent imprinting. These observations demonstrated that the RA-HMMIPs had been successfully synthesized.

The Fourier transform-infrared (FT-IR) spectra of Fe_3_O_4_, Fe_3_O_4_@SiO_2_-RAFT, and RA-HMMIPs are shown in Figure 1b. FT-IR spectra were recorded in the range of 4000–400 cm^−1^. The peak at 583 cm^−1^ is attributed to the stretch of the Fe-O (Curve A) [23]. The spectra image of Fe_3_O_4_@SiO_2_-RAFT showed the characteristic peaks of the Si-O group (812 cm^−1^), Si-O -H group (944 cm^−1^), Si-O-Fe group (460 cm^−1^), and C=S group (1169 cm^−1^) [24]. Especially, the C=S group was formed by the substitution reaction between the benzyl chloride group and the phenyl magnesium bromide, which indicates the RAFT free radical had been successfully formed on the surface of Fe_3_O_4_@SiO_2_; it is mean Fe_3_O_4_@SiO_2_-RAFT tha had been generated [25]. Finally, in the spectra of RA-HMMIPs, the absorption peaks at 1733 cm^−1^, 2950 cm^−1^, and 2990 cm^−1^ are related to the C=O stretching vibration, the C-H stretching vibration of methylene, and the stretching vibration of methyl, respectively [26]. The above indicated that RA-HMMIPs were successfully prepared.

The thermo-gravimetric curves (TGA) of Fe_3_O_4_, Fe_3_O_4_@SiO_2_-RAFT, and RA-HMMIPs are shown in Figure 1c. The Fe_3_O_4_ nanoparticles exhibited high thermostability in the temperature range of 50 °C to 800 °C. The mass loss percentage was 9.3%. It is due to relatively pure magnetic microspheres without impurities. The TGA curves of the Fe_3_O_4_@SiO_2_-RAFT were similar to Fe_3_O_4_ nanoparticles, whose weight percent loss was 14.2%, due to the vaporization of residual water on the surface of magnetic nanoparticles and carbonization of RAFT free radical [25]. For RA-HMMIPs, the mass loss showed a two-step thermal degradation, which can be attributed to the following factors: when the temperature was below 300 °C, the weight loss was primarily due to the vaporization of residual water [27]. In the temperature range of 300~400 °C, the imprinted polymer layer on the Fe_3_O_4_@SiO_2_ surface completely decomposes with significant weight loss (64.9%) [27,28]. In the range of 680~700 °C, a weight loss of 10.6% was observed as a consequence of the thermal decomposition of the structure [29]. In short, The RA-HMMIPs exhibited exceptional thermostability up to 300 °C.

Figure 1d illustrates the magnetic hysteresis loops of RA-HMMIPs. The saturation magnetization value of RA-HMMIP nanoparticles is 19.89 emu/g; the vibrating sample magnetometer (VSM) curve is symmetric about the origin without hysteresis phenomenon, indicating that the polymeric materials have superparamagnetic properties. As illustrated in Figure 1d, under the magnetic field, RA-HMMIPs can be separated from the medium within 30 s. It follows that the magnetic saturation value of RA-HMMIPs was adequate to facilitate the expeditious separation from the solution in the presence of an external magnet, demonstrating a great magnetic response.

### 2.3. Adsorption Behaviors of the RA-HMMIPs

#### 2.3.1. The Performance of Static Adsorption

**Figure 2:**
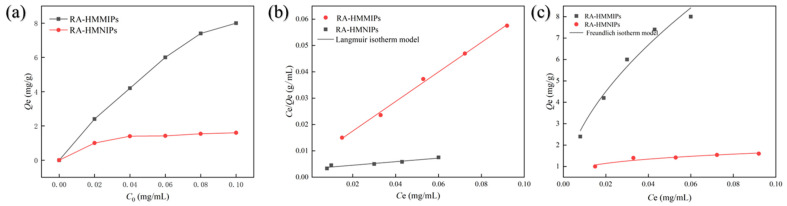
Isotherm models and fitting curves of RA-HMMIPs and RA-HMNIPs. Image (**a**): Static absorption curves. Image (**b**): Langmuir isotherm model fitting curves. Image (**c**): Freundlich isotherm model fitting curves.

The adsorption performances of RA-HMMIPs on different mass concentrations of RA were investigated by static adsorption experiments, while the results are shown in Figure 2a. The adsorption of RA by the polymer increased significantly when the mass concentration was in the range of 2–10 mg/L. The adsorption amount almost did not increase with the further increase in concentration, and it reached the adsorption amount of 8.035 ± 0.37 mg/g (MIPs) and 1.658 ± 0.31 mg/g (NIPs), respectively. Therefore, 10 mg/L concentration of RA was selected in the next experiments. The adsorption amount of NIPs was significantly lower than MIPs, indicating that MIPs have good adsorption capacity. This is due to the fact that the molecularly imprinted layer of RA-HMMIPs has cavities that match the structure of the template molecule, while the MNIPs have no specific sites for RA [30,31].

Furthermore, the aforementioned data were fitted using Langmuir and Freundlich’s isothermal adsorption equations. The fitted parameters of the adsorption models for MIPs and NIPs are presented in Table 2. Among these, the adsorption behaviors that conform to the Langmuir model are characterized by monomolecular-layer adsorption, whereas those that conform to the Freundlich model exhibit multimolecular-layer adsorption. The coefficient of determination (*R*^2^) value of the Freundlich model for RA-HMMIPs (*R*^2^ = 0.973) was higher than that of the Langmuir model (*R*^2^ = 0.892), indicating that the adsorption process involved the formation of a multi-layer. Furthermore, the K_f_ value for RA-HMMIPs was higher than that of RA-HMNIPs, indicating a greater adsorption capacity.

#### 2.3.2. The Performance of Kinetic Adsorption

**Figure 3:**
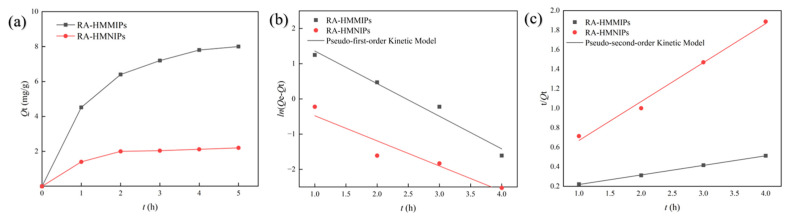
Kinetic adsorption and pseudo-first-order-kinetic model and pseudo-second-order-kinetic model fitting curves of RA-HMMIPs and RA-HMNIPs. Image (**a**): Kinetic adsorption curves. Image (**b**): Pseudo-first-order-kinetic model fitting curves. Image (**c**): Pseudo-second-order-kinetic model fitting curves.

The kinetic adsorption diagrams of RA-HMMIPs and RA-HMNIPs are presented in Figure 3a. The adsorption amount increased rapidly for the first 3 h, then gradually slowed down and reached equilibrium after 5 h, and the adsorption amount of MIPs was 8.012 ± 0.54 mg/g. Because the polymer has many sites that can form hydrogen bonds with RA, it enhances the affinity and accelerates the adsorption process. Once all the sites are filled, the interior of the polymer is the only remaining area for RA adsorption; the interior can only adsorb RA at a low rate. Eventually, all the sites are occupied, and the adsorption process reaches equilibrium. The kinetic adsorption curve of the NIPs exhibited a similar trend to MIPs, with an imprinting factor (IF) of 3.636, which substantiates its efficacy in RA adsorption.

The kinetics of RA-HMMIP adsorption were analyzed by using the pseudo-first-order and the pseudo-second-order models. The image of the fitting model in Figure 3b,c and related parameters is summarized in Table 3. The correlation coefficients (*R*^2^) of the pseudo-second-order kinetic model (*R*^2^ = 0.999) are higher, indicating that the pseudo-second-order kinetics is an appropriate means of describing the adsorption behavior of RA-HMMIPs on RA.

The results show that the adsorption process of RA on MIPs is primarily influenced by the number of unoccupied adsorption cavities on the surface of MIPs, Furthermore, it can be posited that the chemisorption dominates the overall adsorption process.

#### 2.3.3. Selective Adsorption

**Figure 4:**
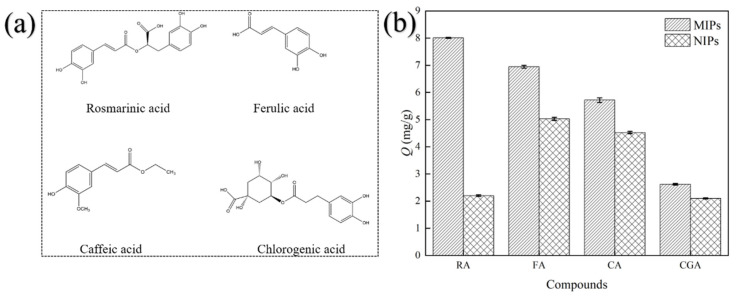
(**a**): The structure of the compounds; (**b**): The selective absorption result of MMIPs.

One of the most significant advantages of MIP technology in comparison to alternative separation techniques is its high degree of selectivity for the template in question, when compared to other compounds of a similar nature and structure. It is based on the generation of specially designed components for the target compound. So, caffeic acid (CA), ferulic acid (FA), and chlorogenic acid (CGA) served as the reference analogs for the selectivity adsorption experiment. The structures of the compounds employed in this essay are illustrated in Figure 4a. Equilibrium adsorption capacities of RA, CA, FA, and CGA on MIP and NIP are shown in Figure 4b, while the selectivity coefficient and imprinting factor are listed in Table 4.

The adsorption capacity of MIPs for RA, CA, FA, and CGA was significantly different. It indicated that MIPs have the specific recognition ability of RA. The adsorption capacity of RA-HMMIPs is highest (8.012 ± 0.54 mg/g) among all other compounds with a similar structural composition. CGA demonstrated a degree of selectivity for MIPs, exhibiting a slightly enhanced adsorption capacity (2.620 ± 0.031 mg/g). However, the adsorption capacity was markedly inferior to that observed for RA. The similar structure between CGA and RA may be a contributing factor to the observed selectivity of MIPs. Conversely, the MIPs demonstrated an acceptable adsorption capacity for CA (6.945 ± 0.052 mg/g) and FA (5.718 ± 0.085 mg/g). Nevertheless, the imprinting factors for both CA (1.38) and FA (1.26) are relatively low, indicating the presence of non-specific interactions in the system. CA is a precursor compound of RA. Furthermore, the selectivity coefficient of MIPs for FA, CA, and CGA is 2.63, 2.88, and 2.91, respectively. The imprinting factor and selectivity coefficient of MIPs for RA are higher than for the other compounds, indicating specific recognition of RA by MIPs. By comparison, the NIPs showed lower adsorption capacity and selectivity RA, which resulted from the absence of a template. In short, the MIPs have higher selective adsorption to RA.

#### 2.3.4. Pore Diameter Distribution Analysis

**Figure 5:**
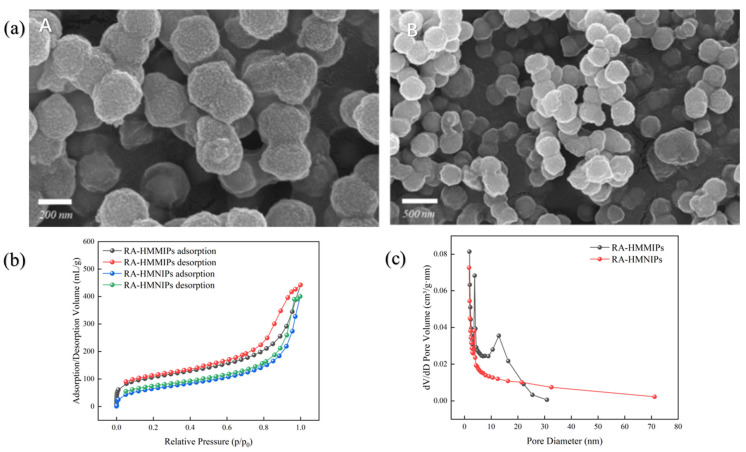
Image (**a**): (**A**) The SEM image of RA- HMMIPs. (**B**) The SEM image of RA- HMNIPs. Image (**b**): the N_2_ adsorption–desorption isotherms curves of MIPs and NIPs. Image (**c**): the Pore diameter distribution curves of MIPs and NIPs.

The scanning electron microscope (SEM) of the polymers was shown in Figure 5a. The surface of MMIPs (A) is rougher than MNIPs (B), which means more specific recognition sites of the RA in the pores. The N_2_ adsorption–desorption isotherms and pore diameter distribution curves of RA-HMMIPs and RA-HMNIPs are shown in Figure 5b. In Figure 5b, the trend of the adsorption and desorption curves of the polymers are the same, but the desorption curves are higher than the absorption curves, which means the polymers are more stable [32]. The hysteresis loop is caused by the accumulation of N_2_ in the pores of the polymer surface. In Figure 5c, the MIPs and NIPs have narrow pore size distributions, and the average pore sizes are small. From the specific surface area and other related data of polymers (Table 5), the pore volume of RA-HMMIPs is 0.820 cm^3^/g, indicating that the structure of the polymer was porous. The specific surface area of RA-HMMIPs is 368.175 m^2^/g, which made it easier for the RA to bind to the sites, speeding up template mass transfer and reducing the equilibrium adsorption time

#### 2.3.5. The Hydrophilic Performance

**Figure 6:**
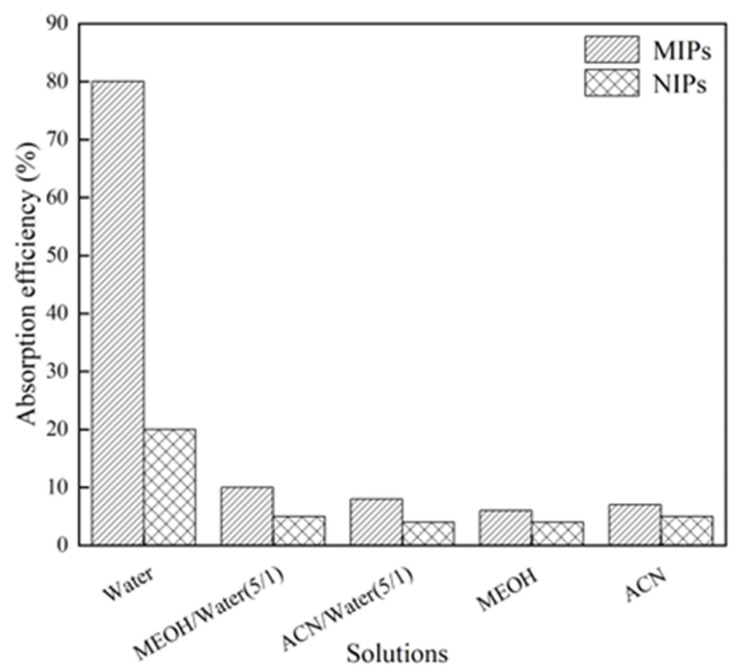
The absorption efficiency of RA on the polymers in different solvents.

The hydrophilic performance of RA-HMMIPs was investigated in water and mixtures: acetonitrile (ACN) to water (5/1), methanol (MEOH) to water (5/1), and ACN and MEOH. As shown in Figure 6, the adsorption efficiency in water was significantly higher than the others, and the adsorption capacity of RA was also higher than that of RA-HMNIPs. These results indicate that the polymer is highly hydrophilic and has more RA sites of action.

### 2.4. Method Evaluation and Real Samples Analysis

**Figure 7:**
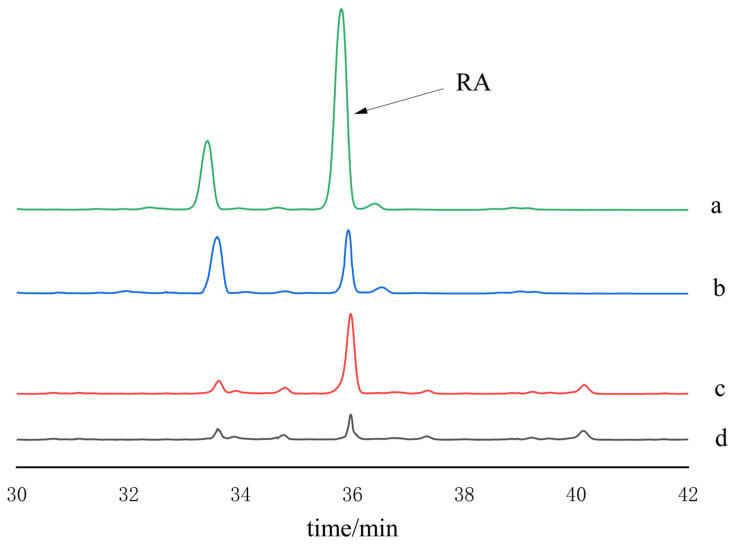
HPLC chromatogram of an extract of *Perilla frutescens* before treating with MIP (a), after treating with MIP (b). *Rosmarinus officinalis* before treating with MIP (c), after treating with MIP (d).

The isolation and enrichment of RA in *Rosmarinus officinalis* and *Perilla frutescensperilla* spiked samples were achieved through the use of HMMIPs. As illustrated in Figure 7a,c, the extract samples were a complex matrix. Following the enrichment by HMMIPs, the amount of RA in the solution was significantly reduced (Figure 7b,d). The results demonstrated that HMMIPs exhibited specific recognition and purification capabilities for RA.

The method exhibits an excellent linear range of 0.8~70 mg/L with correlation coefficients of 0.9988. The LOD (3:1 signal-to-noise)) is 0.27 mg/L. The relative standard deviations (RSD) of the inter-day and intra-day precisions are less than 5% and 4.2%, respectively. The above results show that this method is sensitive, accurate, and suitable for real sample detection.

To assess the feasibility of the method in practice, *Perilla frutescens* and *Rosmarinus officinalis* aqueous extract were analyzed. As presented in Table 6, spiked experiments (20~40 mg/L) further validate the accuracy of the method, resulting in recoveries of 88.2–107.3% with the RSD of 1.3~3.6%. The excellent practicability and accuracy demonstrate that the method has great potential for analyzing RA in aqueous extract.

## 3. Discussion

A number of methods for the extraction and isolation of RA have been reported. To highlight the particular advantages of the method, Table 7 lists other reported research topics on the extraction of RA in plant extracts in recent years, such as the following: soxhlet extraction [33], ultrasound-assisted extraction [34], enzyme-assisted extraction [35], liquid chromatography [36], solid-phase extraction [37], and molecularly imprinted polymer [14]. In comparison to alternative techniques, this method exhibits rapid separation and a high adsorption capacity, while simultaneously adhering to the principles of green chemistry. For example, Mansor et al. [33] used maceration, reflux, and soxhlet as the extraction, with ethanol, 50% aqueous ethanol, and water chosen as the solvents. The reaction time was 6 h. Finally, the highest total extraction yield was obtained from reflux (72.73%) followed by Soxhlet (62.51%) and maceration (37.78%). Jacotet-Navarro et al. [34] investigated ultrasound and microwave extraction with 90% aqueous ethanol (*v*/*v*) to extract the antioxidants from rosemary leaves. The yield was obtained from ultrasound (18.8%) extraction lower than microwave extraction (25.2%). Zhang et al. [36] used ultra-high-performance liquid chromatography coupled with linear ion trap–Orbitrap (UPLC–LTQ–Orbitrap) technology with methanol to evaluate the compounds in *Lonicerae japonicae* Flos. In addition to this, there are some unconventional extraction methods, such as Miron et al. [35] employed an enzyme-assisted extraction method to extract bioactive substances from *Melissa officinalis*, the fundamental principle of which was the degradation of the cell wall through the addition of cellulase and pectinase. And the RA was the predominant ingredient. Feng et al. [14] prepared an acid-resistant magnetic boron-modified molecularly imprinted polymer by the phase transition of lysozyme, which was also used for the selective recognition and separation of RA from *Perilla frutescens* leaf extract with choline amino acid ionic liquid. The adsorption capacity for RA is 6.31 mg/g. From the above reports, most of these techniques suffer from poor selectivity because extraction is based on polarity alone, unlike HMMIPs, which are highly selective for a specific structure over and above the polarity of the compound. Apparently, the current method exhibits higher recoveries and hydrophilicity, demonstrating the feasibility and sensitivity of the current process. Due to the synergetic strategy of the surface imprint method, the constructed HMMIPs are highly hydrophilic and selective and have a low mass transfer resistance, giving the method the advantages of strong anti-interference ability and high detection and mass transfer efficiency.

## 4. Materials and Methods

### 4.1. Chemicals and Reagents

*Perilla frutescens* and *Rosmarinus officinalis* were purchased from markets, Ferricchloride hexahydrate (FeCl_3_·6H_2_O), acetic acid, PEG 2000, ethanol, ethylene glycol, aqueous ammonia, ethyl silicate, methylbenzene, (p-Chloromethyl) phenyltrichlorosilane, triethylamine, acetone, phenylmagnesium bromide solution, tetrahydrofuran, carbon disulfide, ether, acrylamide, N-N′ methylenebisacrylamide, Azodiisobutyronitrile, methanol, acetonitrile, acetic acid, and all the above chemicals were purchased from Aladdin Scientific Corp. (Shanghai, China). Rosmarinic acid (RA), caffeic acid (CA), ferulic acid (FA), and chlorogenic acid (CGA) were purchased from Shanghai yuanye Bio-Technology Co., Ltd. (Shanghai, China).

### 4.2. Preparation of Hydrophilic Magnetic Molecularly Imprinted Polymers

#### 4.2.1. Preparation of Fe_3_O_4_ Nanoparticles

First, 3.0 g of FeCl_3_·6H_2_O, 7.2 g of acetic acid and 3.9 g of PEG 2000 were added to 10.0 mL of ethylene glycol, and the mixture was stirred at 60 °C to fully dissolve. Then, the obtained mixture was heated to 200 °C for 10 h. The black polymer, namely Fe_3_O_4_ nanoparticles, was collected by the magnet and washed 3 times with deionized water and ethanol, then dried under the vacuum for further use.

#### 4.2.2. Preparation of Fe_3_O_4_@SiO_2_

In total, 0.2 g of Fe_3_O_4_ nanoparticles was dispersed in 100 mL ethanol/water (4/1, *v*/*v*) ultrasonically. The mixture was heated to 40 °C with stirring NH_3_·H_2_O (5 mL) and ethyl silicate (2 mL) for 12 h. The product was collected by a magnet and washed three times with deionized water and ethanol, then dried under vacuum for use.

#### 4.2.3. Preparation of Fe_3_O_4_@SiO_2_-RAFT

Overall, 1.0 g of Fe_3_O_4_@SiO_2_, 2 mL of (p-Chloromethyl) phenyltrichlorosilane, and 1 mL of triethylamine was added to 50 mL of methylbenzene under the nitrogen. The system was heated up to reflux for 24 h. The product was washed with acetone three times and dried in storage, and thus named Fe_3_O_4_@SiO_2_-Cl. Then, the Fe_3_O_4_@SiO_2_-Cl was dispersed in the mixture solution contain of 12 mL phenylmagnesium bromide solution, 100 mL tetrahydrofuran, and 4 mL carbon disulfide. The mixture was heated at 60 °C for 48 h under the nitrogen. The product was separated by a magnet, washed by tetrahydrofuran, ether, and ethanol, and vacuum dried for further use, and hence called Fe_3_O_4_@SiO_2_-RAFT.

#### 4.2.4. Preparation of RA-HMMIPs

In total, 0.1 mmoL of RA (dissolved in water) and 0.6 mmoL of AM were added to 50 mL water, which underwent pre-polymerization at 4 °C for 12 h. Then, 0.3 g of Fe_3_O_4_@SiO_2_-RAFT, 20 mmoL of MBA, and 70 mg of ABIN were added to the mixture at 70 °C for 24 h under the nitrogen. The product was collected by a magnet, and washed by methanol/water (8/2, *v*/*v*) and acetic acid/water (9/1, *v*/*v*) in order. The product was vacuum dried, namely in the form of HMMIPs. The non-molecularly imprinted polymers (NIPs) are prepared in the same process as HMMIPs except that RA is not added.

### 4.3. Characterization

The morphological characteristics of the synthesized polymers were observed by JEM-2100F TEM (JEOL Co., Tokyo, Japan) and sigma 500 SEM (ZEISS Co., Oberkochen, Germany). FT-IR spectra were obtained via Nicolet IS 5 FT-IR spectrometers (Thermo Co., Waltham, MA, USA). The magnetic properties were quantified utilizing an LDJ 9600-1 VSM (LDJ Co., Troy, MI, USA). The BET parameter of the nanoparticles was measure by ASAP2020 Version (Micromeritics Co., Norcross, GA, USA). TGA at 10–800 °C was performed on a TGA-55 (TA Instruments Co., New Castle, DE, USA) at a heating rate of 50 °C/min under nitrogen atmosphere.

### 4.4. HPLC Analysis

The determination of RA was performed using an LC-2030 (Shimadzu, Kyoto, Japan). The instrument parameters were as follows: mobile phase, 0.1% methanoic acid/methanol (65:35, *v*/*v*); column temperature, 35 °C; flow rate, 0.9 mL/min; detection wavelength, 330 nm; and injection volume, 10 μL. Samples are filtered through a 0.22 μm membrane prior to analysis.

### 4.5. Adsorption Performance

#### 4.5.1. Static Adsorption

At an initial concentration of 0 to 10 mg/L, the polymers (20 mg) that were obtained in Section 2.2 were dispersed in the aqueous RA solution (4 mL). Following agitation at room temperature for 5 h, the supernatants were separated by magnets and measured by HPLC at 330 nm for calculation of equilibrium concentration of RA. The equilibrium adsorption capacity (*Q*_e_, mg/g) calculation formula is given in Equation (1):

(1) $$Q_{e}=\frac{(C_{0}-C_{e})\times V}{m}$$

Herein, *C*_0_ (mg/mL) is the initial concentration, *C_e_* (mg/mL) is the equilibrium concentration, *V* (mL) is the volume of RA aqueous solution, and *m* (g) is the amount of RA-HMMIPs.

#### 4.5.2. Kinetic Adsorption

A kinetic adsorption study was conducted for determining the influence of adsorption time on adsorption capacity of RA-HMMIPs. The polymers were dispersed in the RA aqueous solution with the concentration of 5 mg/L. Then, the RA concentration in the supernatant was determined by HPLC at 1 h, 2 h, 3 h, 4 h, and 5 h. The adsorption capacity of RA-HMMIPs for RA at each time was calculated by Equation (2).

(2) $$Q_{t}=\frac{(C_{0}-C_{t})\times V}{m}$$

*C*_0_ (mg/mL) is the initial concentration, *C_t_* (mg/mL) is the concentration at different adsorption times, *V* (mL) is the volume of RA aqueous solution, and *m* (g) is the amount of RA-HMMIPs.

#### 4.5.3. Specific Adsorption

The structural analogs of RA, including caffeic acid (CA), ferulic acid (FA), and chlorogenic acid (CGA), were selected as references to investigate the selectivity of RA-HMMIPs. In total, 20 mg of RA-HMMIPs (or NIPs) were added to the 4 mL solution containing RA (dissolved in water), CA (dissolved in ethanol), FA (dissolved in ethanol), and CGA (dissolved in ethanol), respectively. After shaking for 5 h, a supernatant was magnetically separated and HPLC was used to analysis. The selectivity coefficient (*SC*) and imprinting factor (*IF*) were calculated to investigate the selective adsorption of the polymers. The adsorption capacity (*Q*) was calculated by Equation (1); *IF* and *SC* were calculated by the following equations:

(3) $$IF=\frac{Q_{MIPs}}{Q_{NIPs}}$$

(4) $$SC=\frac{IF_{templates}}{IF_{Analogues}}$$

*Q_MIPs_* (mg/g) is the adsorption amount of the MIPs; *Q_NIPs_* (mg/g) is the adsorption amount of the NIPs; *IF_templates_* is the imprinting factor of the RA; *IF_analogues_* is the imprinting factor of the analogue’s compounds

### 4.6. Hydrophilic Adsorption

In total, 20 mg of polymers were dispersed in 4 mL of RA solution at concentration of 10 mg/L. The types of solvents investigated included methanol, acetonitrile, water, methanol/water (5/1, *v*/*v*), and acetonitrile/water (5/1, *v*/*v*). Following the separation of the mixture by magnet at 35 °C for 5 h, the concentration of RA in the supernatant was determined by HPLC. The adsorption efficiency of polymers in different solvents was calculated using Equation (5).

(5) $$\begin{array}{cc} Absorption & efficiency=\frac{m_{Absorption}}{m_{0}}\times 100\% \end{array}$$

*m_Absorption_* (g) is the amount of RA absorption by polymers, and *m*_0_ (g) is the amount of RA in the different solution.

### 4.7. Extraction of RA in Real Samples

#### 4.7.1. Preparation of Samples

*Perilla frutescens* and *Rosmarinus officinalis* were purchased from local supermarkets. The method described in reference was employed for the preparation of the *Perilla frutescens* and *Rosmarinus officinalis* extract and obtained 30 g of dry crude extract [40].

#### 4.7.2. Analytical Performance and Real Sample Analysis

The RA was dissolved by double-distilled water, with a the concentration of 20 to 40 mg/L. Overall, 1.0 g of sample extract was dissolved by 5.0 mL of double-distilled water and 10 mg RA-HMMIPs was added to the solution. After shaking at 40 °C for 5 h, the polymer was separated using a magnet. The samples were washed with 1.0 mL water followed by the desorption of RA using 1.0 mL ethanol. Thereafter, 0.8 mL of the eluate was separated, rotary evaporated, and re-dissolved in 0.6 mL MeOH for HPLC analysis. The calculated results were limit of detection (LOD), spiked recovery, and relative standard deviation (RSD), respectively.

## 5. Conclusions

In summary, RA-HMMIPs were synthesized to extract RA from a real sample in a selective and efficient manner. Compared with the corresponding NIPs and other recently reported methods, the synthesized RA-HMMIPs showed high adsorption capacity and selectivity. And the methods have two key advantages. First, water was used as a porosity-forming agent for synthesizing the polymers, avoids the use of organic solvents, and reduces the influence of interfering substances. Second, the MMIPs exhibit high accuracy, sensitivity and selectivity for RA in aqueous solution. But we should continue exploring advanced methods of MMIPs fabrication.

## Figures and Tables

**Table 1:** Effect of functional monomers on absorption capacity (*Q*).

MIPs	Monomer	Ratio	Adsorption Capacity (mg/g)
1	AM	1:4	3.24 ± 0.25
2	AM	1:6	3.53 ± 0.42
3	AM	1:8	3.35 ± 0.22
4	MAA	1:4	2.78 ± 0.37
5	MAA	1:6	2.68 ± 0.20
6	MAA	1:8	2.76 ± 0.71
7	4-VP	1:4	3.48 ± 0.09
8	4-VP	1:6	3.35 ± 0.93
9	4-VP	1:8	3.40 ± 0.68

**Table 2:** The parameters related to the isotherm models of HMMIPs and HMNIPs.

	Langmuir			Freundlich		
	*Q*_m_ (mg/g)	K_L_	*R* ^2^	n	K_f_	*R* ^2^
RA-HMMIPs	14.850	21.043	0.892	1.756	41.749	0.973
RA-HMNIPs	1.783	88.883	0.996	4.326	2.829	0.912

**Table 3:** The parameters related to the kinetic models of HMMIPs and HMNIPs.

		Pseudo-First-Order Kinetic Model			Pseudo-Second-Order Kinetic Model		
	Q_e,exp_ (mg/g)	Q_e_ (mg/g)	K_1_ (min^−1^)	*R* ^2^	Q_e_ (mg/g)	K_2_ (g·mg^−1^·min^−1^)	*R* ^2^
RA-HMMIPs	8.012 ± 0.54	9.025	0.926	0.972	10.216	0.101	0.999
RA-HMNIPs	2.253 ± 0.29	1.265	0.713	0.909	2.507	2.307	0.987

**Table 4:** The imprinting factor and selection coefficient for RA, CA, FA, and CGA.

Compounds	Imprinting Factor	Selectivity Coefficient
RA	3.64	-
CA	1.38	2.63
FA	1.26	2.88
CGA	1.24	2.91

**Table 5:** Specific surface area and other related data of HMMIPs and HMNIPs.

Samples	S_BET_ (m^2^/g)	Pore Volume (cm^3^/g)	Average Pore Size (nm)
RA-HMMIPs	368.175	0.820	10.036
RA-HMNIPs	326.262	0.690	7.429

**Table 6:** The recovery of RA-HMMIPs on real samples.

Samples	Concentration Range of Spiked (mg/L)	Recovery (%)	RSD (%)
*Rosmarinus officinalis*	20~40	91.2~107.3	1.3~2.5
*Perilla frutescens*	20~40	88.2~99.1	2.1~3.6

**Table 7:** Comparison with the existing methods of RA extraction.

Methods	Plant Source	Extraction Solvent	Absorption Capacity (mg/g)	Recovery	Magnetism	Reference
Maceration, reflux and soxhlet extraction	*Orthosiphon stamineus*	ethanol, 50% aqueous ethanol (*v*/*v*) and water)	-	-	NO	[33]
Ultrasound-assisted Extraction	*Rosmarinus officinalis* L.	90% aqueous ethanol (*v*/*v*)	-	-	NO	[34]
Enzyme-assisted Extraction	*Melissa officinalis*	phosphate–citrate buffer at pH 5.	-	25%	NO	[35]
liquid chromatography	*Lonicerae japonicae* Flos	methanol	-	-	NO	[36]
Carbon paste electrode	*Salvia officinalis*, *Zataria multiflora*, *Mentha longifolia*, *Rosmarinus officinalis*	O-phosphoric acid in water, methanol, 2-propanol	-	92.3~102.9%	YES	[38]
Vacuum microwave-mediated rotary hydrodistillation and extraction	*Perilla frutescens* leaves	choline amino acid ionic liquid	-	99.83%	NO	[39]
Magnetic Molecularly Imprinted Polymer	*Perilla frutescens* leaf	choline amino acid ionic liquid	6.31	Not given	YES	[14]
Magnetic Molecularly Imprinted Polymer	*Rosmarinus officinalis*, *Perilla frutescens*	water	8.012	88.2~107.3%	YES	In this work

-, Not given.

## Data Availability

Data are available on request from the corresponding authors.
